# Role of Microenvironment on the Fate of Disseminating Cancer Stem Cells

**DOI:** 10.3389/fonc.2019.00082

**Published:** 2019-02-21

**Authors:** Vincenzo Ingangi, Michele Minopoli, Concetta Ragone, Maria Letizia Motti, Maria Vincenza Carriero

**Affiliations:** ^1^IRCCS Istituto Nazionale Tumori, Fondazione G. Pascale, Naples, Italy; ^2^Department of Experimental Medicine, University of Campania Luigi Vanvitelli, Naples, Italy; ^3^Department of Sport Science and Wellness, University Parthenope, Naples, Italy

**Keywords:** cancer stem cells, microenvironment, metastatic niche, dormancy, agents targeting microenvironment

## Abstract

Disseminating Cancer Stem Cells (CSCs) initiate growth in specific niches of the host tissues, the cellular and molecular components of which sustain signaling pathways that support their survival, self-renewal dormancy and reactivation. In the metastatic niche, tumor cells may enter in a dormant state to survive and, consequently, the metastasis can remain latent for years. Despite the clinical importance of metastatic latency, little is known about what induces CSCs to enter a dormant state and what allows them to remain viable for years in this state. CSCs exhibit genetic, epigenetic and cellular adaptations that confer resistance to classical therapeutic approaches. The identification of potential CSC targets is complicated by the fact that CSCs may arise as a consequence of their relationship with the local microenvironment into the metastatic niches. Indeed, microenvironment modulates the capability of CSCs to evade the innate immune response and survive. Some new therapeutic options that include drugs targeting microenvironment components are achieving encouraging results in reducing the number of CSCs in tumors and/or overcoming their resistance in preclinical studies. This review will focus on specific CSC features with an emphasis on the role of tumor microenvironment in supporting metastatic dissemination of CSCs. In addition, it sheds light on potential microenvironment-targeted therapies aimed to counteract seeding and survival of CSCs in the metastatic niche.

## Introduction

The development of metastases is a multistep process which involves the detachment of tumor cells from the primary site, their migration and invasion into surrounding stromal tissue, intra-vasation, transit through blood vessels, and extravasation through capillaries (1, 2). The process is followed by colonization of disseminating tumor cells into the so-called metastatic niche within the host tissue (3, 4). Like in the primary niches, cellular and molecular components of metastatic niches regulate survival, and proliferation of tumor cells (3–5). Accumulating evidence document that only a small subset of metastasizing cells are able to persist and to form metastases (6–8). This subpopulation is mainly composed by cancer stem cells (CSCs) that exhibit stem-like properties, are able to grow, invade and self-renewal (9). As in the case of disseminating tumor cells, the capability of CSCs to survive may be due to mutations and it is conceivable that microenvironment forces their genetic evolution toward mutations that favor survival, while less-favorable aberrations leading to cancer cell death are not positively selected. Furthermore, deregulation of various epigenetic pathways involving DNA methylation and chromatin has been shown to contribute to survival of CSCs (10, 11). In this regard, the microenvironment may be considered the promoter of a “clonal” choice that selects those cells able to sustain tumor growth and maintenance (12).

The metastatic niche is a complex network consisting of the vasculature, stromal, inflammatory and immune cells, as well as extracellular matrix (ECM) proteins, signaling, and soluble factors which provide physical anchorage, survival, immune surveillance protection and metabolic requirements (3). Here, tumor cells may enter into G0-G1 arrest to survive and, consequently, the metastasis can remain latent for years. Indeed, dormant tumor cells have been found in patients with prostate cancer (13), melanoma (14), and in breast cancer patients. In the last case, metastasis can occur after decades of an apparent disease-free period (15). Despite the clinical importance of metastatic latency, to date, the molecular mechanisms underlying the capability of CSCs to enter a dormant state and remain viable are not fully elucidated. To develop into an active metastasis, CSCs must have or acquire the ability to exit from dormancy, survive, evade the innate immune response, and initiate proliferation. Tumor microenvironment has been documented to provide signals which regulate self-renewal, epithelial-mesenchymal transition, and homeostatic processes such as inflammation, hypoxia and angiogenesis which regulate either entering of CSCs in a dormant state (dormancy-permissive) either promoting the reactivation of CSCs that initiate metastasis (dormancy-restrictive) (16). To date, the understanding of relationships between circulating tumor cells (CTCs) and circulating CSCs as well as their specific role in determining metastatic dissemination is still debated arising some unresolved issues.

## Pre-Metastatic Niche Formation

Emerging evidence indicates that only a CTC subpopulation mainly constituted by cancer cells with stem-like features displays properties of anchorage-independent survival, and is capable of self-renew, tumor initiation, growth, and dissemination to distant organs (17, 18). To metastasize to distant organs, CTCs have to cross vascular barriers, and some vascular beds are more permissive than others. For instance, fenestrated endothelia of the bone marrow or liver capillaries favor extravasation of many type of solid tumors, including breast and colon cancer (19). Importantly, most of solid tumors spread to lung since thin pulmonary capillaries are adjacent to alveolar cells to allow gas exchange (20). Recently, Liu X. identified a novel mechanism of human breast cancer cluster formation which is mediated by intercellular CD44-CD44 homophilic interactions and dependent on CD44-PAK2 complex-activated downstream pathways that promote cancer stemness and enhance adaptation to microenvironments (21). Kaplan et al. demonstrated for the first time that primary tumors can arrange the microenvironment of distant organs for tumor cell colonization even before their arrival. They found that bone marrow-derived hematopoietic progenitor cells expressing the vascular endothelial growth factor receptor 1 (VEGFR1), home to pre-metastatic niches, and form cellular clusters before the arrival of tumor cells (22). Thereafter, most efforts have been done to investigate the interplay between CSCs and microenvironment to form the metastatic niche. In a recent Review, Liu and Cao described the temporal sequence of the key events occurring during the metastatic niche formation (23).

In the very early stages of formation of an immature pre-metastatic niche, tumor cells actively participate by producing soluble factors, cytokines and chemokines, inflammatory factors that support the pre-metastatic niche formation by recruiting tumor-associated macrophages (TAMs) and neutrophils (TANs), myeloid-derived suppressor cells (MDSCs), and regulatory T (Treg) cells (24, 25). Cancer cells allow a crosstalk with the surrounding components also by producing exosomes and large oncosomes which transfer proteins, mRNAs, microRNAs, small RNAs, and/or DNA fragments into the recipient cells (26–31). For instance, colon-rectal cancer cells promote vascular permeability and angiogenesis in the pre-metastatic niche by transferring exosomal miR-25-3p to endothelial cells (32). Prior to or at the same time of the arrival of CTCs or circulating CSCs in the pre-metastatic niche, the establishment of an hypoxic, inflammatory milieu may help seeding, survival, and proliferation of tumor cells (33, 34). The production and excretion of hydrogen ions, combined with poor perfusion, results in an acidic extracellular pH which is toxic to normal cells, promotes ECM degradation by activating proteinases, increases angiogenesis through the release of VEGF, and inhibits the immune response by stimulating granulocyte colony-stimulating production (35–37). Estrella V. showed that the acidic pH in the tumor microenvironment represents a “niche engineering” strategy that promotes invasion and subsequent *in vivo* growth of malignant tumor cells (38). Accordingly, neutralization of the tumor-derived acidity decreases spontaneous and experimental metastases (39).

The deposition and remodeling of ECM components, including fibronectin, periostin, tenascin-C, collagen IV and lysyl oxidase (LOX) are key processes in the development of the pre-metastatic niche and have been shown to occur before the arrival of tumor cells (40). The hypoxic environment in the pre-metastatic niche regulates gene expression of either collagen, and collagen-modifying enzymes which, in turn, alter collagen structure and organization (41). It has been shown that periostin is required for CSCs maintenance and that CSCs increase the expression of periostin in the fibroblasts of pulmonary pre-metastatic niches (42, 43). Metalloproteinases (MMP)s also plays an important role in organizing the ECM and MMP9 has been shown to recruit bone marrow-derived cells (BMDC) into the pre-metastatic niche (44). Other enzymes, LOX and LOX-like proteins (LOXL), that are upregulated in response to hypoxia, are involved in ECM remodeling during niche formation due to their ability to cross-link collagen and elastin (35–45).

Once recruited by tumor-derived colony-stimulating factor 1 (CSF1), vascular endothelial growth factor A (VEGFA), semaphorin 3A, CC-chemokine ligand 2 (CCL2), and CXC-chemokine ligand 12 (CXCL12), in the pre-metastatic niche, TAMs suppress the cytotoxic activity of CD8^+^ T cells through their expression of programmed cell death 1 ligand 1 (PDL1) and B7-H4 (46, 47). Also, TAMs can indirectly suppress the cytotoxic activity of CD8^+^ T cells through the CCL22-mediated recruitment of Treg cells (48). Dendritic cells (DC)s has a role in orchestrating immune responses (49). Due to their heterogeneity, DCs may switch from an immunostimulatory activation state driving anti-tumor immunity in early stage tumors, to an immunosuppressive activation state at later stages (50, 51). In particular, Kenkel, J.A. identified a DC subset which is responsible to expand Treg and suppress CD8^+^ T cells thereby eliciting an immunosuppressive microenvironment in liver metastasis from pancreatic cancer cells (52). Neutrophils are the main cell population involved in the formation of pre-metastatic niches. Wculek SK demonstrated that neutrophil-derived leukotrienes support lung colonization of metastasis-initiating breast cancer cells by expanding the cancer cells with high tumorigenic potential (53). Furthermore, expansion and polarization of neutrophils promoted by gamma delta (γδ) T cells in the pre-metastatic niche have been shown to favor breast cancer metastasis (54). The occurrence of a relationship between mesenchymal stem cells (MSCs) and CAFs has been described. Once recruited by inflammatory factors within tumor microenvironment, MSCs act as precursors of CAFs which, in turn, contribute to tumor progression by secreting interleukins, chemokines, VEGF, hepatocyte growth factor (HGF), and MMPs (55). Li et al. found that MSCs participate to a cancer stem cell niche formation via release of prostaglandin E2. They found that breast cancer cells elicit induction of the COX-2/microsomal prostaglandin-E synthase-1 axis in MSCs recruited into the pre-metastatic niche by releasing IL-1 which elicits a mesenchymal/stem cell-like phenotype in the breast cancer cells (56). More recently, Su S. demonstrated that a subset of CD10 and GPR77 expressing CAFs, promotes tumor formation and chemoresistance by favoring the formation of a niche for CSCs (57).

## Metastatic Niche Formation

**Figure 1:**
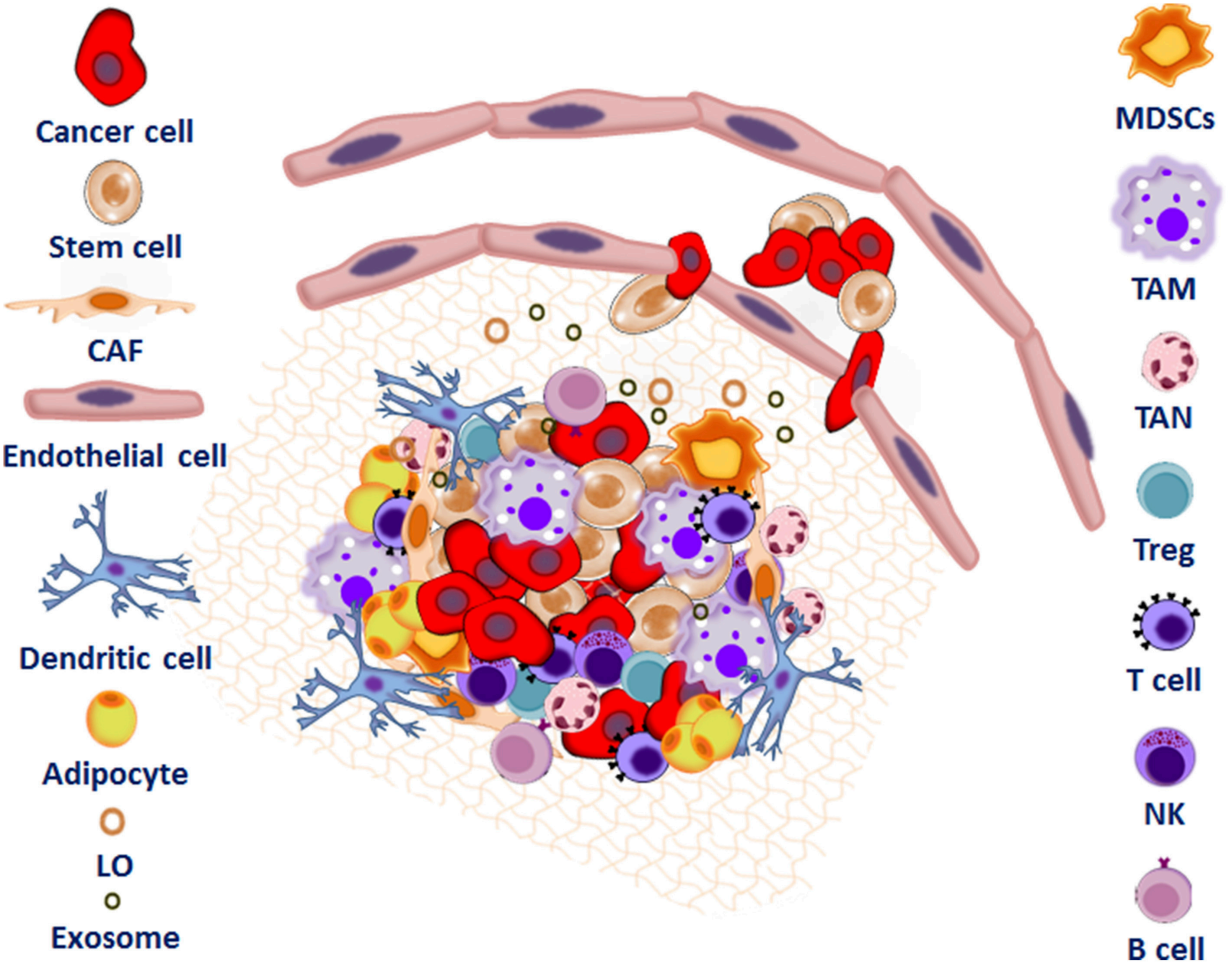
Interplay between tumor microenvironment and cancer stem cells to form the metastatic niche. The metastatic niche is a complex network consisting of the extracellular matrix proteins (ECM), inflammatory and immune cells including tumor associated macrophages (TAMs), regulatory T cells (Treg), myeloid-derived suppressor cells (MDSC), and mesenchymal stem cells (MSC)s. Endothelial cells (EC), adipocytes, cancer associated fibroblasts (CAF), exosomes, microvesicles, and large oncosomes as well as signaling and soluble factors also support tumor cell colonization.

At the end of the priming phase, the establishment of a mature metastatic niche (Figure 1) allows the seeding and colonization of CTCs and/or CSCs. In this phase, CSCs colonize the niche, some of them survive or become dormant until the niche environment becomes suitable to support both seeding and growth of tumor cells, leading to micro-metastases (23). During the progression phase, cells and soluble factors can induce metastatic tumor cells to grow and expand within the niche, leading to macro-metastases (23). Here, adipocyte-rich depots support growth of cancer cells by providing fatty acids, modulating cancer cell metabolism and stemness (39). Recent studies demonstrate that, adipocytes support the survival of prostate and breast cancer cells in the bone metastatic niche though the induction of the oxidative and endoplasmic reticulum stress pathways *via* upregulation of Heme Oxygenase 1 and Survivin (58).

During every step of tumor metastasis, an immunosuppressive behavior within the metastatic niche is indispensable to ensure immunological evasion and consequent tumor cell growth (23). The expansion of immunosuppressive Treg cells as well as MDSCs and macrophages within lymph nodes, liver, lung, and bone marrow have been observed in many types of human cancer (59–61). Furthermore, beside their role in blood vessel formation, endothelial cells regulate tumor growth through the paracrine release of a variety of endothelial-derived growth factors, adhesion molecules such as intercellular adhesion molecule 1 (ICAM1), VCAM1, E-selectin, P-selectin and hyaluronan, and chemokines, such as IL-8, monocyte chemotactic protein 1 (MCP1), and SDF1. By expressing these factors and producing ECM, endothelial cells and endothelial progenitor cells establish a microenvironment which supports the expansion of CSCs (62). Wieland et al. recently showed that the ligand-activated Notch1 receptor on endothelial cell surface induces endothelial cell senescence, expression of chemokines and VCAM1, leading to increased neutrophil infiltration and that the inhibition of Notch1 or VCAM1 reduces metastasis driven by endothelial cells in mouse models (63). Endothelial cells have been also shown to promote CSC phenotype and chemoresistance in colon-rectal cancer cells trough the secretion of the Notch ligand Jagged-1 soluble factor and by activating the cancer stem cell-associated NANOGP8 pathway (64, 65).

Since metastasis often occurs long after the removal of a primary tumor, it is difficult to envision how this progression occurs. Since metastatic CSCs surviving in a dormant state in metastatic niches may explain the timing of metastatic latency, dormancy may be considered an adaptive response to microenvironmental stress (16).

## Microenvironment And CSC Dormancy

Several studies support the notion that metastatic CSCs enter a dormant state by being unable to establish integrin-mediated interactions with the ECM components. Aguirre-Ghiso et al. reported that urokinase plasminogen activator receptor (uPAR) downregulation induces tumor dormancy *in vivo* through the inhibition of the physical interaction of uPA/uPAR complexes with the α5β1 integrin, resulting in lower adhesion of human squamous carcinoma cells to fibronectin and lower MAPK/ERK pathway activation (66). They also found that phosphorylation of p38 forces cells to enter in a quiescence state, whereas a switch toward ERK1/2 activation induces cell proliferation (66). *In vivo*, down-regulation of uPAR in human squamous carcinoma cells inhibited focal adhesion kinase (FAK) phosphorylation and downstream Src activation promoting cellular dormancy (67). The requirement of Src kinase activation in regulating tumor dormancy and metastasis has been documented in breast cancer: Src kinases establish a pro-survival strategy when breast cancer cells were introduced into the bone marrow of nude mice while breast cancer cells died when microenvironment was deprived of Src activity (68). How these dormancy escape mechanisms occur spontaneously in patients and whether they resemble alternative dormancy pathways or cooperate remain to be established.

The TGFβ superfamily is a master regulator of tumorigenesis playing important roles in both promotion and inhibition of cancer cell growth (69). The ligands of TGFβ superfamily activate intracellular pathways either via stimulation of Smad2 and Smad3 for the TGF-β/activin pathway, or Smad1/5/9 for the bone morphogenetic protein (BMP) pathway (70). Several studies identified stromal TGF-β and BMP as inducers of dormancy (71–73). Recently, Mallardi S. documented that TGF-β contributes to the entry of disseminated human lung and breast carcinoma CSCs into a quiescent state through the down-regulation of MYC (74). In this study, they show that quiescent tumor cells evade surveillance and elimination of metastatic seeds by NK cells and that Sex determining region Y-box 2 (SOX2) and SOX9 transcription factors are essential for CSCs survival and metastatic outgrowth (74).

Other factors have been shown to induce dormancy of cancer cells in the metastatic niches of bone. For example, the morphogenetic protein 7 (BMP7) has been documented to induce dormancy of prostate cancer cells. The treatment of mice with BMP7 significantly induced senescence in CSCs suppressing their growth in bone, whereas BMP7 removal restarted growth of CSCs (73). Price TT. showed that the CXCL2/CXCR4 interaction binds breast cancer cells to microenvironment of the bone marrow and that CXCR4 inhibition prevents metastatic progression (75). Tumor dormancy is not only the result of cancer cells undergoing cellular quiescence but may also be caused by a reduced vascularization (angiogenic dormancy) or by the cytotoxic activity of immune system (immune mediated-dormancy). Angiogenic dormancy refers to the period when the factors that inhibit endothelial cell proliferation and vessel sprouting predominate, leading to oxygen and nutrient deprivation (16, 76).

An angiogenic switch can be induced by unbalancing the levels of pro-angiogenic factors, such as VEGF or platelet-derived growth factor (PDGF) and anti-angiogenic factors, including angiostatin, endostatin, and thrombospondin-1 (2–4). As a result, there is a blockage of tumor growth and cells remain in a quiescent state. Also, dormant tumor cells may be unable to remodeling preexisting vasculature, leading to hypoxia and limited nutrient supply. Alternatively, micro-metastases may escape dormancy because of down-regulation of circulating angiogenesis inhibitors (77).

Immune surveillance controlling tumor dormancy utilizes immune pathways very similar to those active during tumor destruction in the elimination phase, mostly including cytotoxic effector/memory T cells, and Th1 cytokines (78). However, tumor dormancy implies that the tumor cells have “survived” to the elimination phase and that their progression may be successfully restrained by immune-mediated mechanisms reflecting activation of Th1-associated factors such as interferon-γ (IFN-γ), IL-12, and up-regulation of CD8 genes (78, 79). An interruption of this equilibrium will advantage only dormant tumor cells bearing genetic or epigenetic changes which will favor their entering in a proliferative state (80).

## Targeting Microenvironment To Prevent Growth and Dissemination Of CSCs

**Figure 2:**
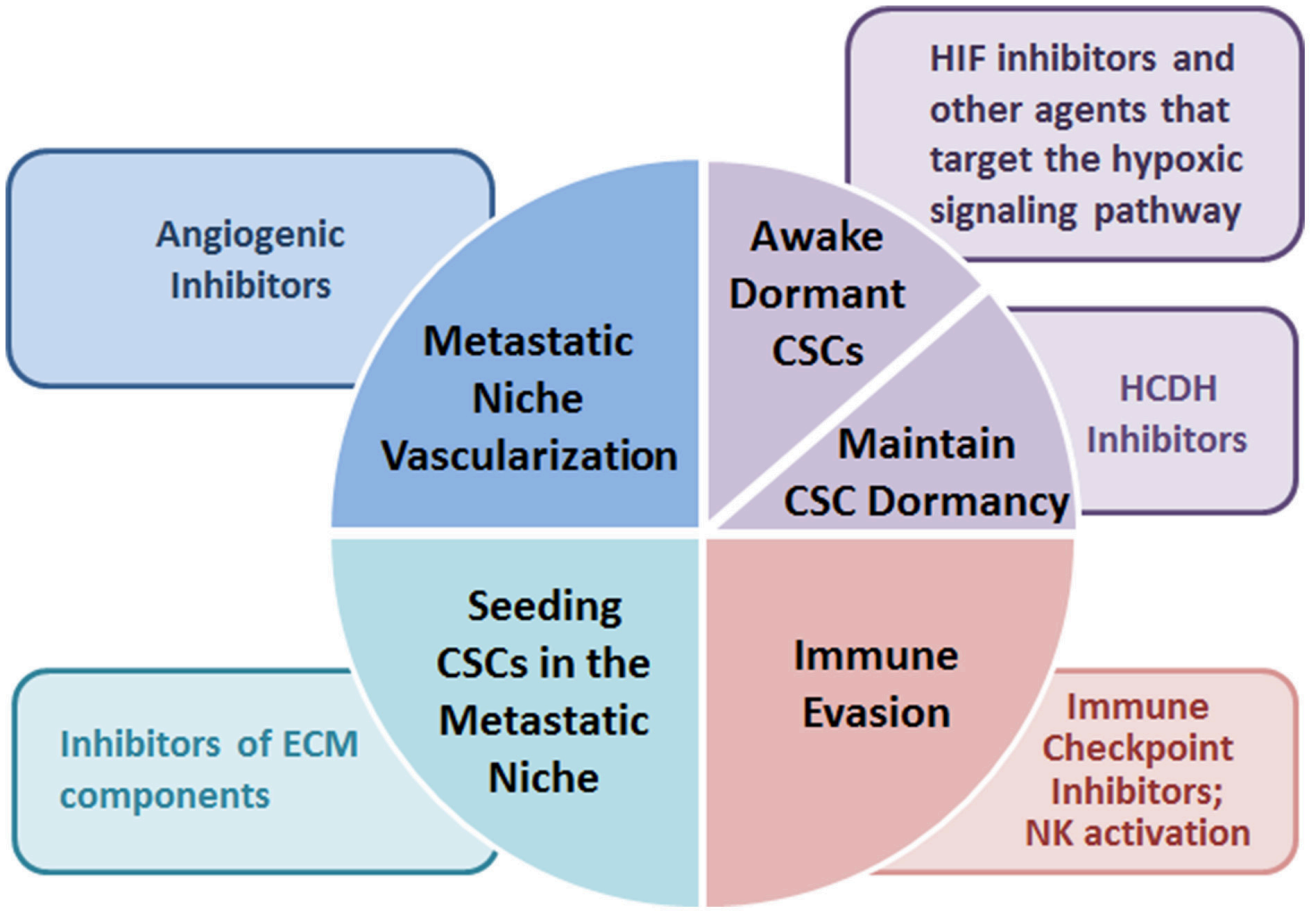
Potential microenvironment-targeted therapies. Microenvironment-targeted therapies suggested for preventing metastatic dissemination of cancer stem cells include agents modifying deposition and remodeling of ECM components, drugs counteracting the hypoxic signaling pathways, angiogenic inhibitors, and immune checkpoint inhibitors converting the immunosuppressive behavior to an immunostimulatory one.

Identification of potential CSC targets is complicated by the fact that CSCs may arise as a consequence of their relationship with cellular and soluble components of the microenvironment that affect their capability to evade the innate immune response and survive. Thus, attempting to deprive CSCs of microenvironmental support may allow the development of new therapeutic strategies aimed to prevent growth and dissemination of CSCs (Figure 2).

For example, ECM components, are emerging attractive targets for preventing the seeding of CSCs in the pre-metastatic niche and combinatorial therapies which include inhibitors of LOX, MMPs, Tenascin C or Periostin have been suggested in preclinical models (81). Anti-angiogenic therapies might reduce the proportion of CSCs in different tumors, thus being a valuable therapeutic approach to eradicate resistant and aggressive tumor cells. However, this approach has obtained contradictory results. The VEGF-specific antibody bevacizumab reduces metastatic niche formation in rectal carcinoma patients (82) and its combination with an anti-hepatoma-derived growth factor antibody impairs CSCs, preventing tumor relapse and progression in non-small cell lung cancer hetero-transplant tumor models (83). Conversely, the administration of bevacizumab combined to VEGF receptor tyrosine kinase inhibitor sunitinib induces tumor hypoxia in breast cancer cell lines that increases the CSC population (84). Anti-angiogenic drugs often induce tumor hypoxia, allowing CSCs to survive and propagate, thus driving tumor progression. In this regard, De Francesco E. recently suggested that doxycycline, an inhibitor of mitochondrial biogenesis, increases the sensitivity of hypoxic breast cancer cells to conventional chemotherapies, such as paclitaxel, overcoming hypoxia-induced drug-resistance *in vitro* (85). A possible explanation for the failure of angiogenic inhibitors may be due to the angiogenic dormancy: inhibition of endothelial cell proliferation and vessel sprouting elicits oxygen and nutrient deprivation which allows CSCs to enter in a dormant state (16–76). In this regard, a variety of Hypoxia-inducible factors (HIF) inhibitors and other agents that target the hypoxic signaling pathway are in preclinical and clinical development for cancer (86). Other hypoxic response target genes that have been linked to metastatic niche formation in xenograft models include SDF-1α, TGF-β, MMP-2, MMP-9, and CXCR4 (87). An alternative possibility could be maintain, rather than awake, dormant CSCs. This is the case of histone deacetylase inhibitors (HDACi) that prolong dormancy and drive tumor cells into a differentiated, quiescent state of melanoma cells in murine models (88). Furthermore, CSCs often acquire resistance to anti-angiogenic therapy, mainly due to their genomic and epigenetic heterogeneity that increases expression of drug transporters and DNA damage repair capability (89).

Cancer immuno-therapy is a novel anti-cancer strategy with a recent increasing success and emerging data suggest that immune checkpoint inhibitors may be successfully employed for the eradication of CSCs in tumors. CSCs seem to have a unique immune evasion features that include the overexpression of PD-1/PD-L1 molecules and subsets of CSCs expressing the CTLA4 ligand B7.2 and/or PD-1 have been found in solid tumors (90, 91). In the context of the immune escape by CSC, a possible strategy to overcome CSC resistance to NK-induced killing is to modulate NK receptor expression in melanoma cells in order to elicit NK-mediated immune response against CSCs. Indeed, CSCs are highly susceptible to NK cell-mediated killing thus suggesting that NK cell-based immunotherapy might be successfully employed for the treatment of patients with colon cancer (92). These preclinical studies highlight the challenges of interpreting data involving signals from the tumor microenvironment, as they can be pleiotropic and involve multiple cell types.

## Concluding Remarks

Despite the clinical importance of metastatic latency, to date, the molecular mechanisms underlying the ability of CSCs to enter a dormant state and remain viable for years are not fully elucidated. CSCs are resistant to conventional treatments and there is emerging evidence that CSCs can acquire resistance to anti-angiogenic therapy and/or evade novel immunogenic therapies. Thus, understanding the mechanisms driving pre-metastatic niche formation or underlying the metastatic dormancy process could help to maintain patients in a controlled or disease-free state. To this end, new models aimed to determine what CSC-supporting pathways are important before, during, and after the latency phase of metastasis could allow the develop new pharmaceutical agents capable to eliminate or differentiate CSCs.

## Author Contributions

MVC and VI conception of the work. MM, CR, and MLM extensive literature search and manuscript drafting. VI, MM, and MVC critical revision of the work. MVC final version approval.

### Conflict of Interest Statement

The authors declare that the research was conducted in the absence of any commercial or financial relationships that could be construed as a potential conflict of interest.
